# Mesenchymal stem cell - derived extracellular vesicles modulate immune function in sepsis

**DOI:** 10.3389/fimmu.2026.1881925

**Published:** 2026-07-14

**Authors:** Qinghe Meng, Yuanhui Song, Chunyan Wang, Adam Novak, Niitiggya Taneja, Zhen Ma, Alex Helkin, Robert N. Cooney

**Affiliations:** 1 Departments of Surgery, State University of New York (SUNY), Upstate Medical University, Syracuse, NY, United States; 2 Department of Biomedical & Chemical Engineering, Syracuse University, Syracuse, NY, United States

**Keywords:** apoptosis, cytokines, extracellular vesicles, immunosuppression, iMSC-EVs, peripheral blood mononuclear cell (PBMC), sepsis

## Abstract

**Background:**

Severe sepsis is characterized by dysregulated immune responses associated with high mortality. Both hyperinflammation and progressive immunosuppression contribute to poor outcomes, yet reliable biomarkers and effective immunomodulatory therapies remain limited. Extracellular vesicles derived from induced pluripotent stem cell-derived mesenchymal stem cells (iMSC-EVs) represent a promising cell-free therapeutic strategy, but their effects on immune dysfunction in human sepsis are poorly characterized.

**Methods:**

Fifty-three patients with surgical sepsis meeting Sepsis-3 criteria were prospectively enrolled. Sixty-day mortality was used to define the survivor (n=26) and non-survivor (n=27) groups. Logistic regression identified mediators independently associated with mortality and SOFA score. Cytokine profiles in plasma and media were quantified by ELISA. Peripheral blood mononuclear cells (PBMCs) were isolated and treated with LPS, iMSC-EVs or both. Apoptosis, caspase-3 expression, and acute-phase markers (CRP, SAA) were assessed.

**Results:**

Increased age, cancer diagnosis and SOFA score were associated with mortality. Non-survivors exhibited significantly elevated IL-10, MCP-1, and IL-10/TNF-α ratio, with a lower IL-6/IL-10 ratio. IL-10 (OR = 1.12), MCP-1 (OR = 1.05), and IL-10/TNF-α ratio (OR = 6.61) independently predicted 60-day mortality. LPS-stimulated PBMCs from non-survivors showed a distinct pattern of cytokine responses compared to survivors. iMSC-EV treatment attenuated LPS-induced inflammation, reduced the IL-10/TNF-α ratio, decreased apoptosis and caspase-3 activation, and modulated CRP and SAA in a context-dependent manner.

**Conclusions:**

Patient characteristics and cytokine levels were associated with 60-day sepsis mortality and reflect immunosuppression severity. iMSC-EVs exert immunomodulatory and cytoprotective effects on septic patient-derived PBMCs, supporting their potential as a cell-free therapeutic strategy for restoring immune homeostasis in sepsis.

## 1 Introduction

Sepsis, a life-threatening syndrome triggered by a dysregulated immune response to infection, remains a leading cause of death in intensive care units (ICUs), with mortality rates ranging from 20–50% (1). In many patients sepsis progresses through a hyperinflammatory phase, characterized by a cytokine storm and excessive immune activation, followed by a prolonged immunosuppressive phase marked by immune cell dysfunction, apoptosis, and increased susceptibility to secondary infections (2). Both phases contribute to poor prognosis, with hyperinflammation driving early organ dysfunction and immunosuppression leading to late-stage complications and higher mortality (3). The dysregulated host immune response in sepsis is orchestrated by a complex network of innate and adaptive immune effector cells, each contributing distinct cytokine profiles that collectively drive the pathological inflammatory cascade. Monocytes and macrophages serve as the primary producers of pro-inflammatory cytokines, including TNF-α, IL-1β, and IL-6, following pattern recognition receptor activation by pathogen-associated molecular patterns such as LPS, while simultaneously producing IL-10 as a counter-regulatory mediator to limit excessive tissue injury (4). Elevated pro-inflammatory cytokines, such as IL-6, TNF-α, IL-8, and monocyte chemoattractant protein-1 (MCP-1), dominate the hyperinflammatory phase, while persistent lymphopenia, reduced monocyte HLA-DR expression, and elevated IL-10 levels are hallmarks of immunosuppression, strongly associated with increased mortality risk (5–9).

IL-10, an anti-inflammatory cytokine, exacerbates immunosuppression by suppressing pro-inflammatory cytokine production, inhibiting T-cell proliferation, and downregulating antigen-presenting cell function, predisposing patients to secondary infections (4, 10–13). In severe sepsis, persistent IL-10 overproduction and a high IL-10:TNF-α ratio were strongly associated with mortality, while elevated TNF-α, IL-6, IL-1ra, and sTNFR predicted early hemodynamic deterioration (14). Also, IL-10/lymphocyte ratio level is significantly associated with disease severity and prognosis in patients with severe sepsis (15). The IL-10/TNF-α and IL-6/IL-10 ratios serve as critical indicators of the balance between pro- and anti-inflammatory responses, with higher IL-10/TNF-α and lower IL-6/IL-10 ratios reflecting a shift toward immunosuppression and correlating with worse outcomes (16, 17). Additionally, low levels of interleukin-7 (IL-7), essential for immune cell activation and survival, further impair immune recovery during immunosuppression, correlating with increased mortality (18, 19).

Mesenchymal stem cell-derived extracellular vesicles (MSC-EVs) have emerged as a promising therapeutic strategy to modulate both hyperinflammatory and immunosuppressive phases of sepsis while promoting tissue repair (20). These nano-sized vesicles deliver bioactive cargo, including proteins, lipids, microRNAs (miRNA), and other nucleic acids, which exert anti-inflammatory, immunomodulatory, and regenerative effects on target cells (21, 22). Immunomodulatory miRNAs (e.g., miR-146a, miR-223) have been shown to suppress hyperinflammation by inhibiting NF-κB and NLRP3 activation (23, 24). In sepsis, MSC-EVs attenuate excessive inflammation by inhibiting pro-inflammatory signaling pathways and reducing the secretion of cytokines like IL-6, TNF-α, IL-8, and MCP-1, thus mitigating the cytokine storm and associated organ damage (25–28). Concurrently, MSC-EVs counteract immunosuppression by restoring immune cell function and balancing cytokine profiles (29, 30). Evidence from animal studies supports the therapeutic potential of MSC-EVs in sepsis by reducing mortality via decreasing pro-inflammatory cytokines (IL-6, TNF-α), increasing IL-10 to modulate early inflammation, and enhancing macrophage phagocytosis and neutrophil bacterial killing, thereby improving immune responses and reducing organ injury (27, 31, 32). Several clinical trials, including a Phase III study employing bone marrow-derived MSC-EVs, are currently evaluating the safety and efficacy of MSC-EVs for treating sepsis and ARDS (33–37).

MSC-EVs can be sourced from multiple tissue origins, including bone marrow, adipose tissue, and umbilical cord, each with distinct proteome and RNA cargo profiles. Although early findings are promising, these studies are limited by a lack of standardization regarding MSC-EV source, biologic heterogeneity, therapeutic efficacy and safety, scale-up/manufacturing, storage and stability and cargo variability (38, 39). Consequently, reported effects on disease biomarkers, therapeutic efficacy, and clinical outcomes remain inconsistent. MSCs derived from human induced pluripotent stem cells (iPSCs) represent a promising strategy to overcome the limitations associated with primary MSC sources, including donor variability, limited proliferative capacity, and scalability constraints (40–43). MSCs generated from human induced pluripotent stem cells (iMSCs) (44–46) are expected to provide a reproducible and sustainable cell source for therapeutic applications. We have developed a robust serum-free differentiation protocol that enables consistent generation of iMSCs through an intermediate neural crest stage (47–49). This streamlined differentiation strategy, combined with modular manufacturing processes and serum-free culture conditions, facilitates scalable and standardized production of both iMSCs and their extracellular vesicles (EVs). Importantly, lineage-specific iMSCs exhibit distinct transcriptomic profiles and differential EV production capacity compared to primary tissue-derived MSCs, underscoring the importance of iMSC source specification when interpreting therapeutic outcomes (49).

Our lab has shown iMSCs exhibit expression profiles of MSC-specific biomarkers comparable to those of primary MSCs (49). Moreover, iMSCs derived EVs (iMSC-EVs) attenuate pulmonary inflammation in murine models of lung injury, reduce systemic inflammation and improve survival in a murine endotoxemia model (50). Based on these findings, the objective of the present study is to identify biomarkers associated with mortality in sepsis and to determine whether iMSC-EVs can restore immune homeostasis and functional responses in peripheral blood mononuclear cells (PBMCs) isolated from septic patients.

## 2 Materials and methods

### 2.1 Study population

This study was approved by the Institutional Review Board of SUNY Upstate Medical University (IRB protocol #1790554-5). Adult patients (≥18 years) with suspected or confirmed infection were screened for enrollment using the Sepsis-3 criteria (1). Patients with a qSOFA score ≥2 (altered mental status, systolic blood pressure ≤100 mmHg, or respiratory rate ≥22 breaths/min) underwent full SOFA score assessment. The SOFA score was calculated based on the following parameters: PaO_2_/FiO_2_ ratio, Glasgow Coma Scale (GCS) score, mean arterial pressure (51), vasopressor requirement (including agent, dose, and infusion rate), serum creatinine and urine output, serum bilirubin level, and platelet count. Patients with surgical sepsis, including those with gastrointestinal perforation or intestinal ischemia requiring operative intervention, were considered eligible for enrollment. Potential participants were identified by surgery residents, midlevel providers, and attending surgeons within the Trauma, Surgical Critical Care, and Acute Care Surgery services at Upstate Medical University. Patients were excluded if a patient or family member was unable to give consent or consent was withheld. Do not resuscitate (DNR) status was not considered an exclusion to participation. Several patients were transitioned to comfort care measures only, typically when their condition worsened despite aggressive source control and supportive measures and they continued to deteriorate clinically. The patient screening, enrollment, and allocation process is outlined in the flowchart shown in **Supplementary Figure 1**.

Clinical data, including patient demographics, clinical outcomes, and SOFA scores, were collected from 53 patients who met the diagnostic criteria for Sepsis-3 from 2019 to 2025 at surgical ICU of Upstate Medical University. Upon meeting the preliminary screening criteria, informed consent was obtained on the same day. 20 ml of peripheral blood was collected from indwelling vascular catheters, immediately placed on ice to mitigate cellular stress under *ex vivo* hypoxic conditions and transported to the laboratory for processing. Plasma was isolated by centrifugation for subsequent quantification of inflammatory mediators and downstream experimental analyses.

All patients received management consistent with the Surviving Sepsis Campaign guidelines. Standard care comprised prompt surgical source control, including abdominal drainage, surgical debridement, bowel resection, and abscess drainage as clinically indicated, followed by broad-spectrum antimicrobial therapy with subsequent de-escalation guided by microbiological culture results. Hemodynamic support included protocolized fluid resuscitation and vasopressor administration when required. Organ support measures, such as mechanical ventilation for respiratory failure and renal replacement therapy for acute kidney injury, were instituted according to clinical necessity. Post-surgical complications, including anastomotic leak, surgical site infection, intra-abdominal abscess, and secondary bacteremia, were managed with re-intervention or escalation of antimicrobial therapy as appropriate and recorded as clinical outcomes.

### 2.2 Isolation and treatment of peripheral blood mononuclear cells

PBMCs were isolated from whole blood using density-gradient centrifugation. Briefly, peripheral blood samples were collected in EDTA anticoagulant tubes and diluted 1:1 with sterile PBS. The diluted blood was carefully layered onto Ficoll-Paque PLUS (Cat. 17144003, Cytiva) in 50 mL conical tubes and centrifuged at 400 × g for 30 min at room temperature with the brake turned off. After centrifugation, the PBMC layer located at the plasma - Ficoll interface was carefully aspirated and transferred to a new tube. Cells were washed twice with PBS by centrifugation at 300×g for 10 min to remove residual Ficoll and platelets. The final cell pellet was resuspended in appropriate culture medium or PBS. Cell viability and concentration were determined using a hemocytometer. Isolated PBMCs were either used immediately for downstream assays or cryopreserved in freezing medium containing 10% DMSO in fetal bovine serum and then stored in liquid nitrogen for future use.

PBMCs were seeded in 6-well plates at a density of 1 × 10^6^ cells per well in 2 mL of complete medium. After an overnight recovery period, cells were treated with exosomes at the indicated concentrations (4 μg/mL) and incubated at 37 °C in a humidified atmosphere containing 5% CO_2_. For inflammatory stimulation studies, PBMCs were exposed to lipopolysaccharide (LPS, 1µg/mL) in the presence or absence of exosomes. Following 24 hours of treatment, culture supernatants were collected for cytokine analysis by ELISA, and cells were cytospun onto glass microscope slides at 300 rpm for 3 minutes using a cytocentrifuge for immunofluorescence staining.

### 2.3 Generation, isolation, and characterization of iMSC-derived extracellular vesicles

Human induced pluripotent stem cells (hiPSCs) were differentiated into mesenchymal stem cells (iMSCs) through a neural crest intermediate stage as previously described (47, 49, 50). Briefly, hiPSCs were maintained on Geltrex-coated plates in Essential 8 medium (Cat. A1517001, Life Technologies) and sequentially induced toward neural crest lineage using Essential 6 medium (Cat. A1516401, Life Technologies) supplemented with bFGF, SB431542, and the WNT agonist CHIR99021. Neural crest cells were subsequently differentiated into iMSCs in StemPro MSC serum-free medium for approximately 18 days. For EV production, iMSCs at 70 - 80% confluence was cultured in serum-free DMEM supplemented with a cell stimulation cocktail to enhance vesicle secretion. Conditioned media were collected and subjected to sequential processing to isolate EVs. First, samples were cleared of cells and debris by low-speed centrifugation, followed by concentration using 100 kDa molecular weight cut-off ultrafiltration. EVs were then enriched using a commercial precipitation-based isolation reagent and subsequently purified by size-exclusion chromatography (SEC) to improve purity and reduce co-isolated soluble protein contaminants. The resulting iMSC-derived EVs (iMSC-EVs) were characterized using complementary approaches, including cryogenic electron microscopy (cryo-EM) to assess vesicle morphology, dynamic light scattering (DLS) to determine size distribution, and Western blot analysis for EV-associated markers, including CD9, CD63, CD81, and HSP90α/β. EV protein concentration was quantified using a BCA assay and used for experimental dosing.

For *in vitro* uptake studies, PBMCs were incubated with fluorescently labeled iMSC-EVs using the ExoGlow™ Protein EV Labeling Kit (Green, Cat. EXOGP300A-1, SBI) for 4 h at 37 °C. Cells were then washed twice with PBS to remove unbound EVs. EV internalization was visualized using fluorescence microscopy (Nikon TE2000-U, Nikon, Melville, NY). Efficient internalization of iMSC-EVs by PBMCs was demonstrated by fluorescence imaging, as shown in **Supplementary Figure 2**.

### 2.4 Immunofluorescence staining

Immunofluorescence staining was performed to evaluate protein localization in cultured cells. Samples were washed with phosphate-buffered saline (PBS) and fixed in 4% paraformaldehyde (Cat. 19943, USB) for 15 min at room temperature. After fixation, samples were washed three times with PBS and permeabilized with 0.1% Triton X-100 in PBS for 10 min. Non-specific binding was blocked by incubation with 5% bovine serum albumin (BSA) in PBS for 1 h at room temperature. Samples were then incubated overnight at 4 °C with primary antibodies diluted in blocking buffer. Primary antibodies (Serum amyloid A: Cat. Sc-59679 1:50, C-reactive protein Cat. 69770 1:50) were purchased from Stanta Cruz. After primary antibody incubation, samples were washed three times with PBS and incubated with appropriate fluorophore-conjugated secondary antibodies (Cat. AB181289, 1:500, Abcam) for 1 h at room temperature in the dark. Nuclei were counterstained with DAPI (Cat. AB104139, Abcam). Fluorescence images were acquired using a fluorescence microscope under identical exposure settings for all experimental groups. For quantitative analysis, four randomly selected fields per sample were imaged. Each experimental condition included 3 independent biological replicates. Fluorescence intensity was quantified using ImageJ software, and background fluorescence was subtracted prior to analysis. All image acquisition and quantification were performed in a blinded manner to minimize bias. Fluorescence intensity and localization were analyzed using ImageJ software.

### 2.5 Enzyme-linked immunosorbent assay

Cytokine levels were quantified using an enzyme-linked immunosorbent assay (ELISA) according to the manufacturer’s guidelines. Absorbance (OD) was measured at 450 nm using a microplate reader. All kits were purchased from Thermo Fisher Scientific (TNF-α: Cat. 88-7346-88, IL-1β: Cat. BMS224-2HS, IL-6: Cat. 88-7066-88, IL-10: Cat. 88-7106-88, IL-1α: Cat. BMS243-2, IL-1Rα: Cat. BMS2080, IL-7: Cat. EHIL7, IL-8: Cat. KHC0081, IL-17α: Cat. BMS2017, IL-18: Cat BMS267-2, MCP-1: Cat. BMS281, MIP-1α: Cat. KAC2201, MIP-1β: Cat. BMS2030INST).

### 2.6 TUNEL assay

Apoptotic cell death was assessed using a TUNEL assay with the Click-iT Plus TUNEL Assay Kit (Cat. #: C10618 & C10617, Invitrogen) according to the manufacturer’s instructions. Briefly, cultured cells were fixed with 4% paraformaldehyde (Cat. 19943, USB) for 15 min at room temperature and washed three times with PBS. Samples were then permeabilized with 0.1% Triton X-100 in PBS for 10 min. Following permeabilization, samples were incubated with the TdT reaction mixture containing terminal deoxynucleotidyl transferase (TdT) and modified nucleotides for 60 min at 37 °C to label DNA strand breaks associated with apoptosis. After washing with PBS, samples were incubated with the Click-iT reaction cocktail to fluorescently label incorporated nucleotides. Nuclei were counterstained with DAPI (Cat. AB104139, Abcam) and visualized using a fluorescence microscope. TUNEL-positive cells were identified by red (C10618) and green (C10617) fluorescence and quantified by counting positive cells in randomly selected microscopic fields using ImageJ software. The percentage of apoptotic cells was calculated as the number of TUNEL-positive nuclei relative to the total number of nuclei stained with DAPI.

### 2.7 Statistical analyses

Statistical analyses were performed using GraphPad Prism and SPSS. Data are presented as mean ± standard error of the mean (SEM) for continuous variables and as counts or percentages for categorical variables. Normality of data distribution was assessed using the Shapiro–Wilk test and the Kolmogorov–Smirnov test. Comparisons between two groups were performed using an unpaired Student’s t-test for normally distributed data or the Mann–Whitney U test for non-normally distributed data. Comparisons among multiple groups were analyzed using one-way analysis of variance followed by Bonferroni correction. For non-parametric multiple-group comparisons, the Kruskal–Wallis test followed by Dunn’s multiple comparisons test was used.

To evaluate the association between inflammatory mediators and 60-day mortality, logistic regression analysis was performed to calculate odds ratios and 95% confidence intervals, with adjustment for potential confounders including age, sex, and cancer. In addition, multiple linear regression was used to assess the relationship between inflammatory mediators and disease severity as measured by the SOFA. A *P* value < 0.05 was considered statistically significant.

## 3 Results

### 3.1 Baseline characteristics of the study population

A total of 53 patients who met the Sepsis-3 diagnostic criteria were included in the analysis, including 26 survivors (49.1%) and 27 non-survivors (50.9%) at 60 days. Baseline characteristics of the study population are summarized in **Table 1**. There was no significant difference in gender distribution between the two groups (*P* > 0.05). However, non-survivors were significantly older than survivors (70.4 ± 11.7 vs. 54.8 ± 15.17 years, *P* < 0.01). The prevalence of cancer comorbidity was also significantly higher in the non-survival group compared with survivors (*P* < 0.01). Disease severity assessed by the SOFA was significantly higher in non-survivors than in survivors (9.8 ± 2.9 vs. 8.1 ± 2.3, *P* < 0.05), indicating more severe organ dysfunction in patients who died.

**Table 1:** Baseline characteristics of patients in the survival and non-survival groups.

Characteristic (mean ± SD or %)	Survival (n=26, 49.1%)	Non-survival (60-day) (n=27, 50.9%)	Total (n=53)	Ratio (survival: non-survival)	P value survival vs. non-survival
Gender	Male: 18 Female: 8	Male: 17 Female: 10	Male:35 Female: 18	Male: 1.1 Female: 0.8	>0.05 >0.05
Age (year)	54.8 ± 15.17	70.4 ± 11.7	62.8 ± 15.5	0.78	<0.01
Cancer	4 (30.7)	9 (69.3)	13	0.44	<0.01
Survival time (Day)		20.45 ± 32.5			
SOFA	8.1 ± 2.3, n=26	9.8 ± 2.9, n=27	9.0 ± 2.7, n=53	0.82	<0.05
SII	3871.2 ± 3968.5, n=23	2964.8 ± 2754.3, n=19	7417.9 ± 26170.2, n=43	1.31	>0.05
PLT	234.2 ± 149.6, n=25	191.0 ± 116.4, n=21	214.5 ± 135.8, n=46	1.23	>0.05
PMN	11.5 ± 5.7, n=24	11.0 ± 7.0, n=20	113. ± 6.2, n=44	1.05	>0.05
Lymph	0.76 ± 0.43, n=24	0.87 ± 0.54, n=20	0.81 ± 0.48, n=44	0.87	>0.05
IL-1α	30.5 ± 42.5, n=10	43.4 ± 42.6, n=11	37.2 ± 41.9, n=21	0.70	>0.05
IL-1β	8.5 ± 13.3, n=10	26.4 ± 31.1, n=11	17.9 ± 25.4, n=21	0.32	0.055
IL-1RA	3790.4 ± 4078.0, n=10	3438.5 ± 4630.7, n=11	3606.1 ± 4728.4, n=21	1.10	>0.05
IL-6	2440.6 ± 4111.57, n=25	3334.5 ± 5742.37, n=27	2904.8 ± 4996.94, n=52	0.73	>0.05
IL-7	10.9 ± 17.0, n=10	2.1 ± 1.53, n=11	6.3 ± 12.31, n=21	5.19	0.051
IL-8	554.6 ± 830.1, n=10	1052.6 ± 1805.1, n=11	815.5 ± 1415.7, n=21	0.53	>0.05
IL-10	122.3 ± 244.45, n=25	493.6 ± 756.50, n=27	315.1 ± 595.80, n=52	0.25	<0.05
IL-17α	17.8 ± 29.1, n=9	15.4 ± 13.1, n=11	16.5 ± 21.2, n=20	1.16	>0.05
IL-18	290.7 ± 184.0, n=9	227.5 ± 192.4, n=11	255.9 ± 186.5, n=20	1.28	>0.05
MCP-1	1657.8 ± 1252.00, n=24	3314.9 ± 3109.71, n=27	2535.1 ± 2539.19, n=51	0.50	<0.05
MIP-1α	33.2 ± 53.6, n=9	36.6 ± 37.1, n=11	35.1 ± 43.9, n=20	0.91	>0.05
MIP-1β	182.3 ± 160.0, n=9	181.2 ± 202.5, n=11	181.7 ± 19.8, n=20	1.01	>0.05
TNF-α	426.1 ± 229.39, n=25	974.9 ± 2158.37, n=27	711.0 ± 1576.65, n=52	0.44	>0.05
IL-10/TNF-α	0.30 ± 0.32, n=25	1.65 ± 3.16, n=27	1.01 ± 2.36, n=52	0.18	=0.037
IL-6/IL-10	26.9 ± 23.84	11.6 ± 9.67, n=27	19.0 ± 19.37, n=52	2.31	=0.005
PMN/Lymph	33.9 ± 64.3, n=23	14.6 ± 10.5, n=20	24.9 ± 48.1, n=43	2.32	>0.05

SII, Systemic Immune-Inflammation Index; PLT, Platelet; PMN, polymorphonuclear neutrophil; Lymph, Lymphocyte.

### 3.2 Cytokine/Chemokine levels in survivors and non-survivors

Several inflammatory mediators differed between the survival and non-survival groups (**Table 1**). Anti-inflammatory cytokine IL-10 levels were significantly higher in non-survivors (*P* < 0.05). In contrast, the IL-6/IL-10 ratio was significantly lower in non-survivors (*P* = 0.005), suggesting an altered inflammatory balance associated with poor outcomes. Chemokine monocyte chemoattractant protein-1 (MCP-1) levels were also significantly higher in the non-survival group (*P* < 0.05). Additionally, the IL-10/TNF-α ratio was significantly increased in non-survivors (*P* = 0.037). Other inflammatory mediators, including IL-6, TNF-α, IL-8, and IL-1β, did not show statistically significant differences between groups.

### 3.3 Logistic regression analysis of mortality

Multivariable logistic regression analysis adjusted for age, gender, and cancer demonstrated that several inflammatory mediators were independently associated with 60-day mortality (**Table 2**). Elevated IL-10 (OR = 1.12, *P* = 0.049) and MCP-1 (OR = 1.05, *P* = 0.047) were significantly associated with increased mortality risk. In addition, a higher IL-10/TNF-α ratio was strongly associated with mortality (OR = 6.61, *P* = 0.028). Conversely, the IL-6/IL-10 ratio was inversely associated with mortality (OR = 0.898, *P* = 0.007). No significant associations were observed for IL-6 or TNF-α individually.

**Table 2:** Association of inflammatory mediators on 60-day mortality -Logistic regression analysis.

Variables	OR	95% CI	P value
IL-6	0.87	0.983 - 1.071	>0.05
IL-10	1.12	1.010 - 1.125	0.049
MCP-1	1.05	1.00 - 1.008	0.047
TNF-α	1.001	0.999 - 1.003	>0.05
IL-10/TNF- α	6.608	1.229 - 35.530	0.028
IL-6/IL-10	0.898	0.831 - 0.972	0.007

Adjusted by Gender, age and cancer.

### 3.4 Association between inflammatory mediators and disease severity

Multiple variable analysis examining associations between inflammatory mediators and SOFA score revealed that IL-10, MCP-1, and the IL-10/TNF-α ratio were positively associated with disease severity (*P* < 0.05) (**Table 3**). In contrast, the IL-6/IL-10 ratio was negatively associated with SOFA score (*P* = 0.041). These findings suggest that dysregulation of pro- and anti-inflammatory cytokine balance is associated with increased organ dysfunction in septic patients.

**Table 3:** Association of inflammatory mediators on SOFA mortality -multiple variable analysis.

Variables	Standardized coefficient	95% CI	P value
IL-6	0.227	0 - 0.02	0.13
IL-10	0.477	0.01 - 0.03	<0.01
MCP-1	0.371	0-0.01	0.012
TNF-α	0.174	0 - 0.01	0.255
IL-10/TNF- α	0.466	0.234 - 0.857	<0.01
IL-6/IL-10	-0.297	(-0.83) - (-0.22)	0.041

Adjusted by Gender, age and cancer.

### 3.5 Effects of LPS on PBMC cytokine levels

**Figure 1:**
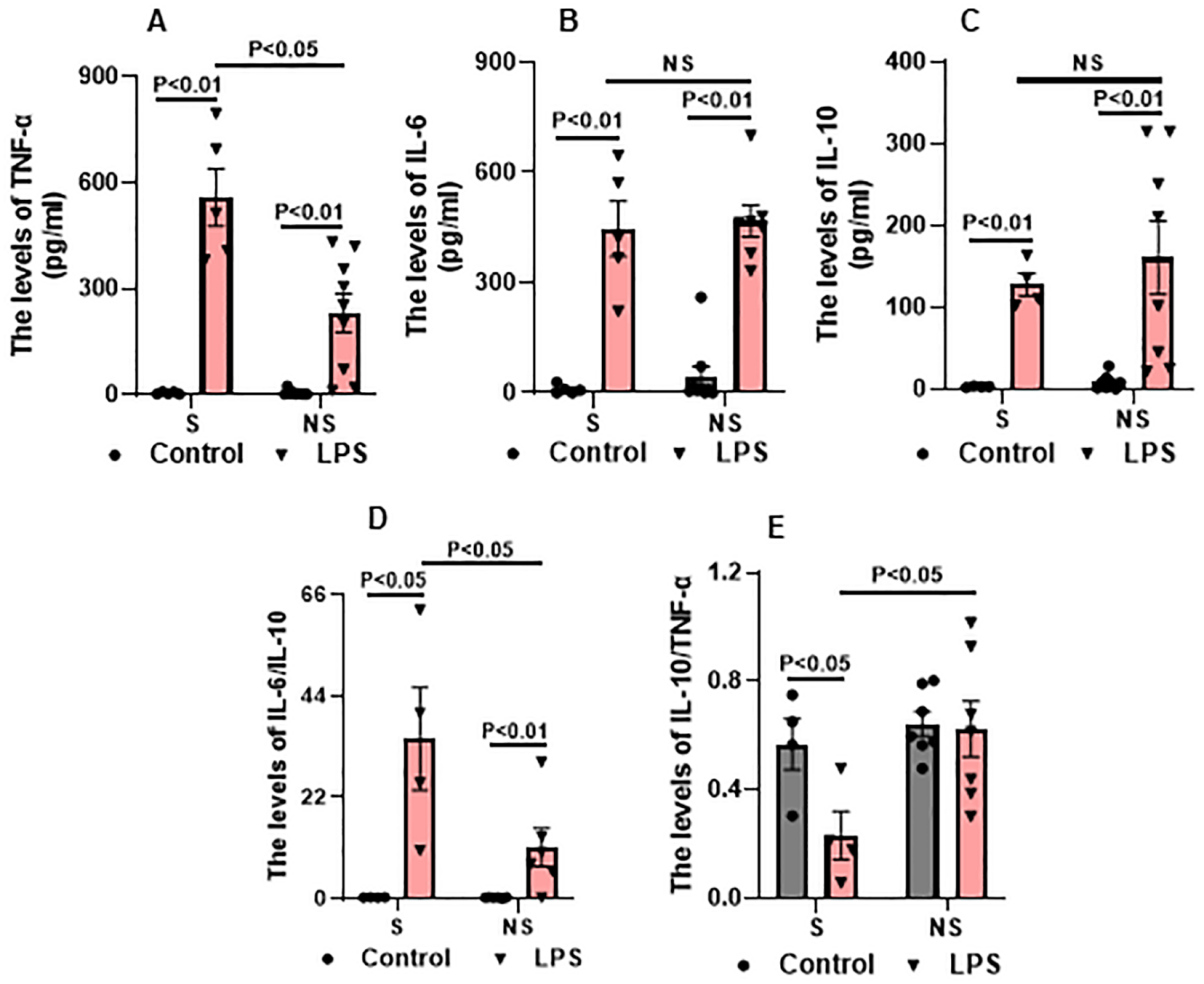
LPS stimulates inflammatory responses in PBMCs: PBMCs from survivors and non-survivors were treated either with LPS (1µg/ml) or PBS for 24 h. The culture medium was collected for the examinations of TNF-α **(A)**, IL-6 **(B)** and IL-10 **(C)** using ELISA. IL-6 to IL-10 **(D)** and IL-10 to TNF-α **(E)** were calculated. Scatter bar graph represents mean values and standard error of mean (SE) (n=4-8 / group). S: Survival, NS: Non-Survival.

Peripheral blood mononuclear cells (PMBCs) were isolated and treated with LPS to assess activation of the Toll-like receptor 4 (TLR4) and NF-κB signaling pathway mediated cytokine production by these cells. Cytokine levels before and after LPS-stimulation of PMBCs from the survivor and non-survivor groups are shown in **Figure 1**. LPS stimulation significantly increased TNF, IL-6 and IL-10 levels in PBMCs from both groups compared with untreated controls (**Figure 1**). Of note, PBMCs derived from survivors demonstrated a more pronounced TNF response to LPS than those from non-survivors (**Figure 1A**). In contrast, LPS-induced changes in IL-6 and IL-10 were not significantly different (**Figures 1B. C**). LPS-stimulated PBMCs from non-survivors exhibited a lower IL-6/IL-10 ratio (**Figure 1D**) and higher IL-10/TNF-α ratio (**Figure 1E**). These data suggest the PMBC fraction (predominantly lymphocytes and monocytes) from non-survivors is “less responsive” to LPS stimulation in terms of inflammatory cytokine production.

### 3.6 Effects of iMSC-EVs on PBMC cytokine levels

**Figure 2:**
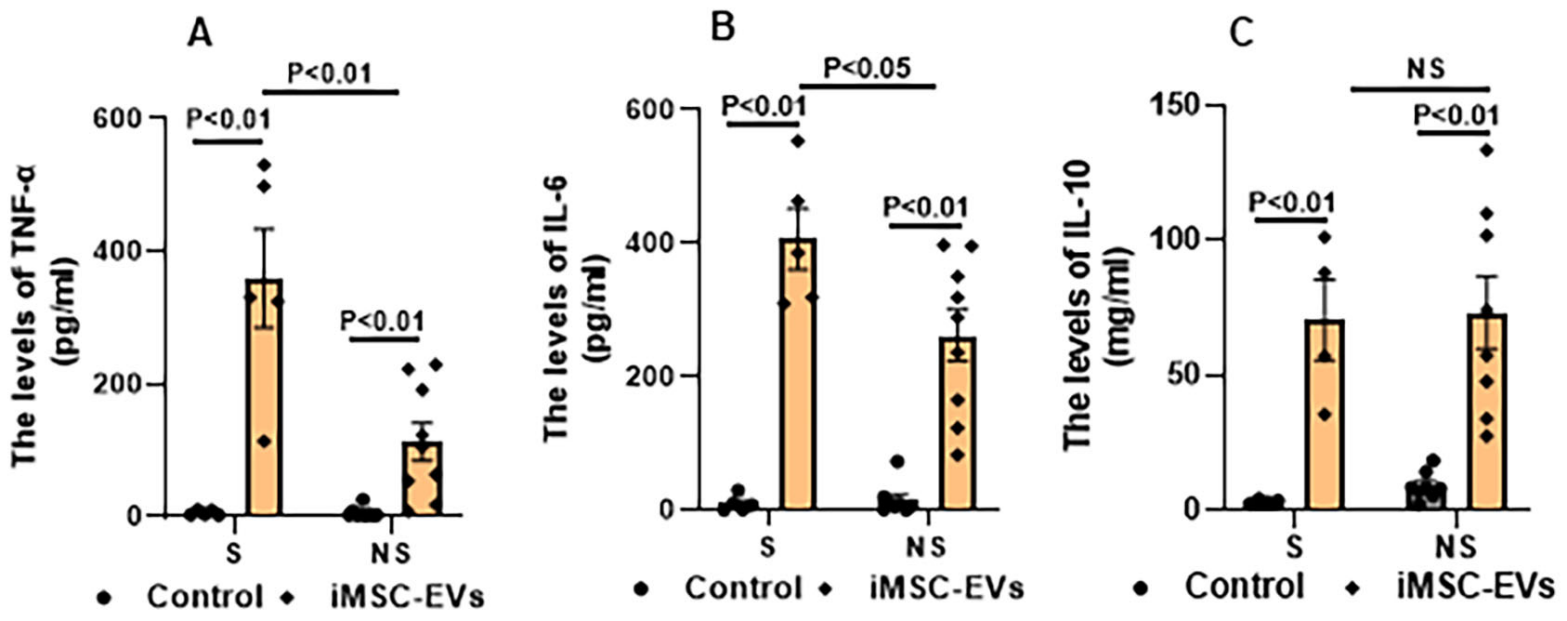
iMSC-EVs activates inflammatory responses in PBMCs: PBMCs from survivors and non-survivors were treated either with iMSC-EVs (4 µg/ml) or PBS for 24 h. The culture medium was collected for the examinations of TNF-α **(A)**, IL-6 **(B)** and IL-10 **(C)** using ELISA. Scatter bar graph represents mean values and standard error of mean (SE) (n=4-8/group). S, Survival; NS, Non-Survival.

Treatment of PMBCs with iMSC-EVs increased TNF and IL-6 in both groups (**Figures 2A, B**), but to a lesser extent in non-survivors. In contrast, IL-10 levels in PMBCs treated with iMSC-EVs were increased to a similar extent in both groups (**Figure 2C**). These findings suggest that iMSC-EVs possess immunomodulatory properties capable of regulating immune responses in septic patient-derived PBMCs, indicating that PBMCs from survivors may be more responsive to the immunomodulatory effects of iMSC-EVs, whereas immune cells from non-survivors exhibit a relatively blunted response.

### 3.7 iMSC-EVs modulate LPS-induced cytokines in PMBCs

**Figure 3:**
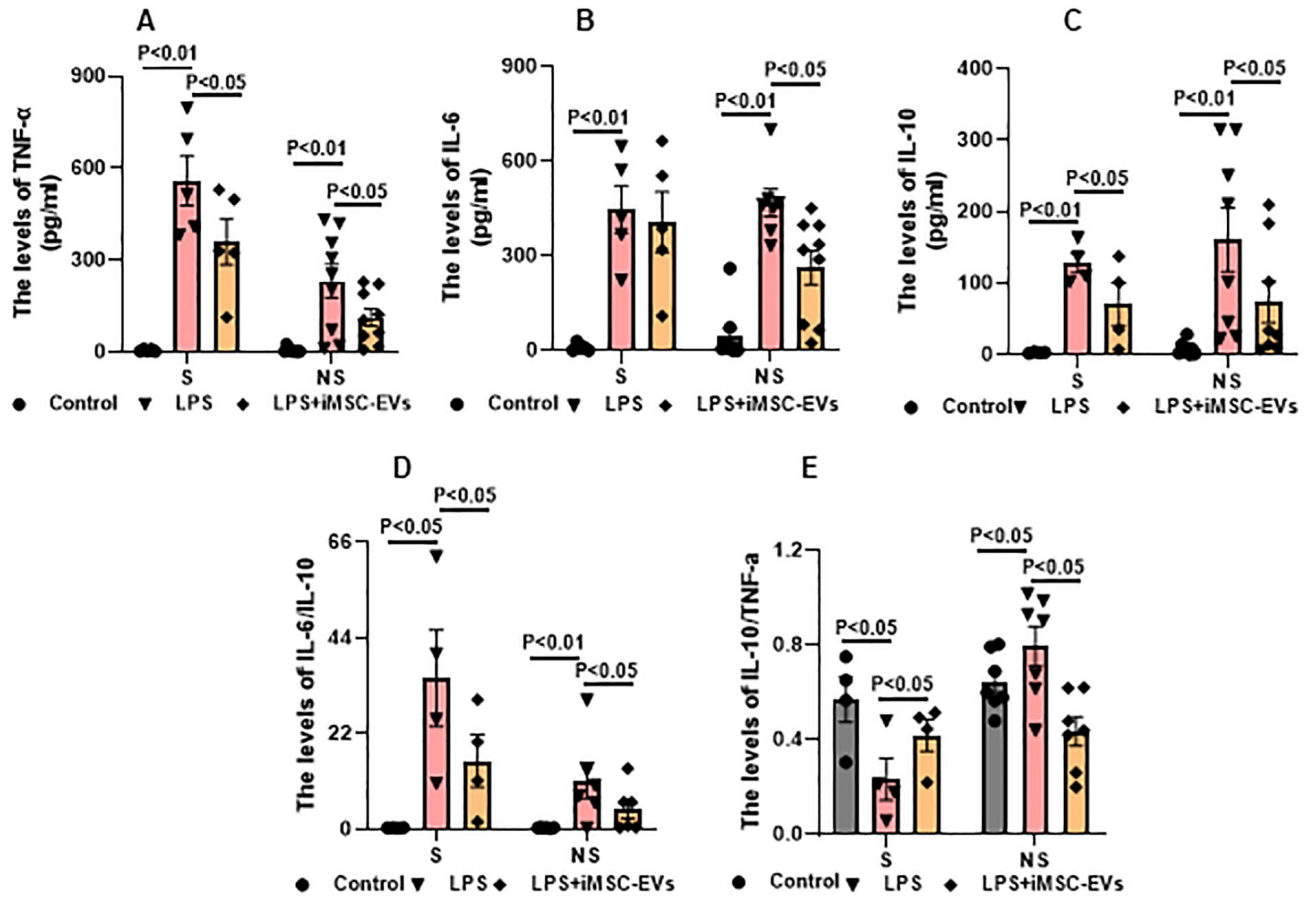
Effects of LPS and iMSC-EVs on immune dysregulation in PBMCs: PBMCs from survivors and non-survivors were treated either with LPS (1µg/ml) ± iMSC-EVs (4 µg/ml) or PBS for 24 h. The culture medium was collected for the examinations of TNF-α **(A)**, IL-6 **(B)** and IL-10 **(C)** using ELISA. IL-6 to IL-10 **(E)** and IL-10 **(D)** to TNF-α **(E)** were calculated. Scatter bar graph represents mean values and standard error of mean (SE) (n=4-8/group). S, Survival; NS, Non-Survival.

To determine whether iMSC-EVs could alter LPS-induced inflammation PMBCs from septic patients were treated with both LPS and iMSC-EVs (**Figure 3**). iMSC-EVs reduced LPS-induced TNF and IL-10 in both survivors and non-survivors, but only IL-6 in non-survivors (**Figures 3A–C**). iMSC-EVs reduced the LPS-induced IL-6/IL-10 levels in both groups, but only the IL-10/TNF ratio levels in PMBCs from non-survivors (**Figures 3D, E**).

Co-treatment significantly attenuated LPS-induced inflammatory responses compared with LPS stimulation alone excluding IL-6 in the survival group (**Figures 3A–D**, *P* < 0.05), indicating that iMSC-EVs can partially restore immune balance under inflammatory conditions. Notably, the IL-10/TNF-α ratio decreased following iMSC-EV treatment in PBMCs from non-survivors (**Figure 3E**), suggesting a shift toward a more pro-inflammatory phenotype and potential reversal of sepsis-associated immunosuppression.

### 3.8 iMSC-EVs reduce apoptosis in PBMCs

**Figure 4:**
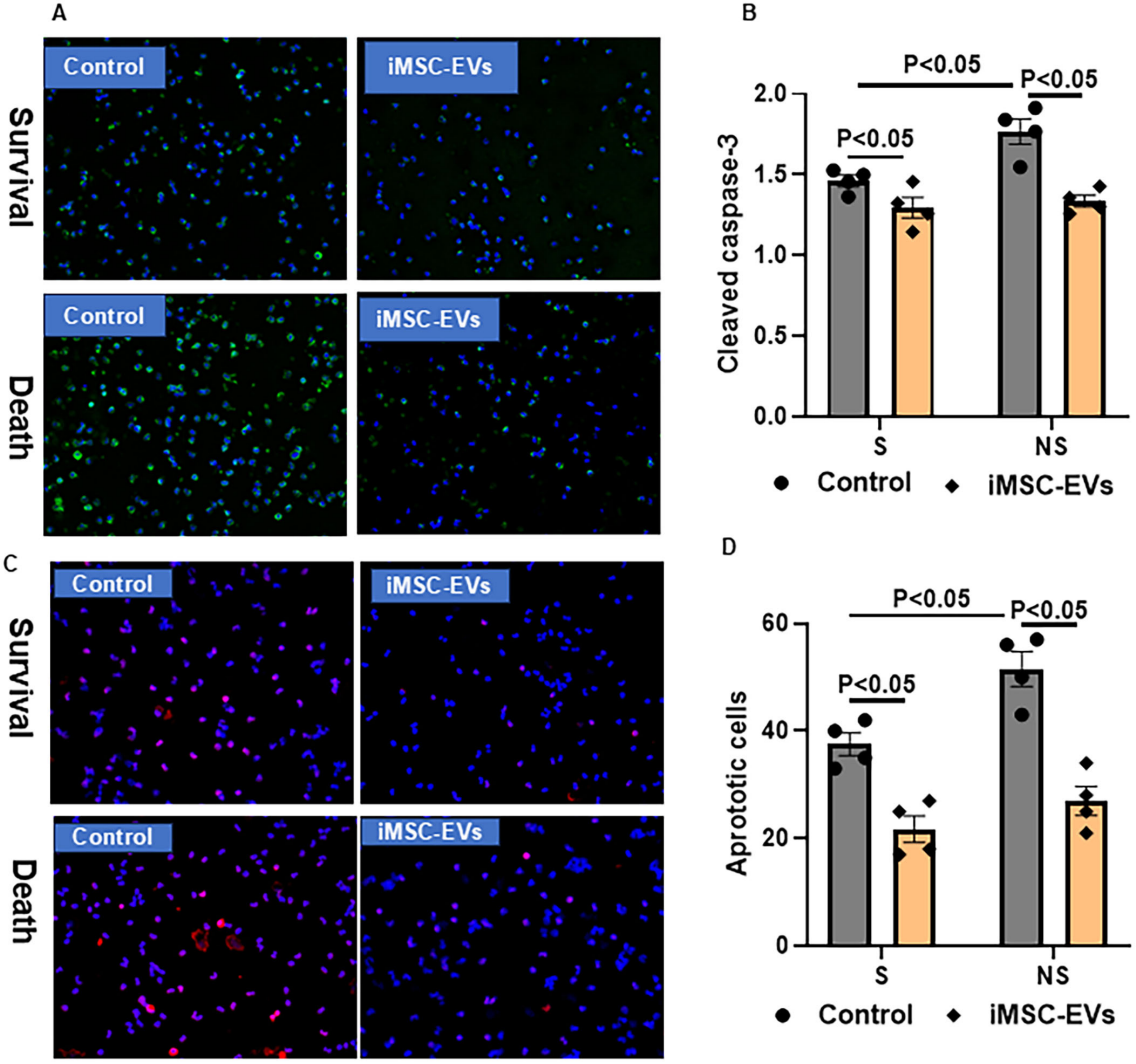
Effect of iMSC-EVs on apoptosis in PBMCs: PBMCs from survivors and non-survivors were treated either with iMSC-EVs (4 µg/ml) or PBS for 24 h. The PBMCs were assayed for cleaved caspase-3 protein **(A)** by immunofluorescence staining and apoptotic cell **(B)** by TUNEL. Scatter bar graph represents mean values and standard error of mean (SE) (n=4/group). S, Survival; NS, Non-Survival.

Apoptosis of immune cells is another important aspect of sepsis-induced immune suppression. To determine if iMSC-EVs impact apoptosis in PBMCs we assayed cleaved caspase-3 protein by immunofluorescence and apoptotic cells by TUNEL assay (**Figure 4**). Apoptosis and cleaved caspase-3 protein were present in all sepsis patients but were significantly increased in the non-survivor PMBCs (**Figure 4**, P<0.05 vs. survivors). Treatment of PMBCs with iMSC-EVs significantly reduced the number of apoptotic cells and the expression of the pro-apoptotic protein cleaved caspase-3 in PBMCs from both survivor and non-survivor groups (**Figure 4**, *P* < 0.05). These findings suggest that iMSC-EVs exert cytoprotective effects on PMBCs from septic patients, attenuating apoptosis and potentially preserving immune cell viability during septic inflammation.

### 3.9 iMSC-EVs regulate acute phase proteins in PMBCs

**Figure 5:**
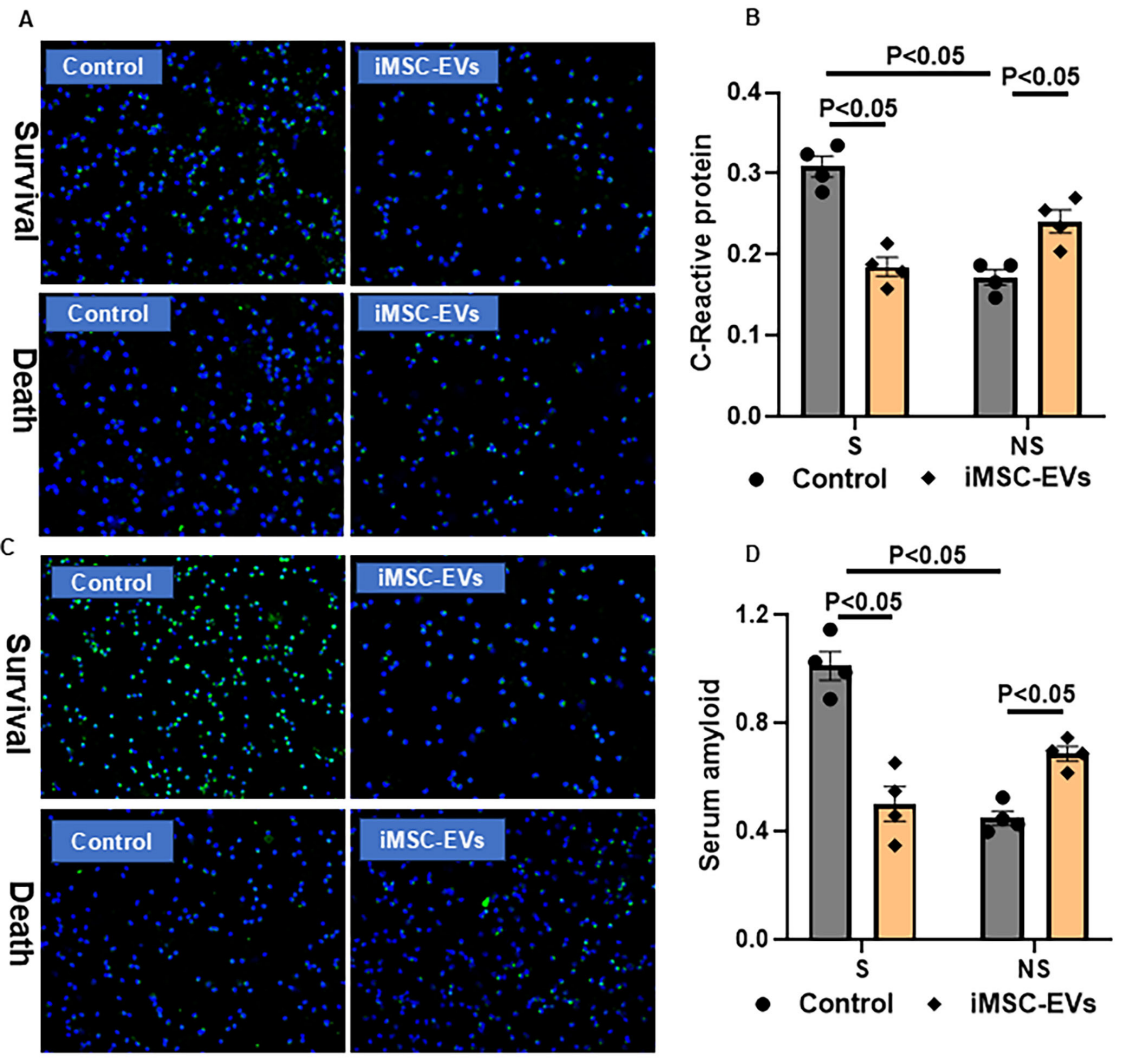
Effects of iMSC-EVs on C-reactive protein (CRP) and serum amyloid A (52) in PBMCs: PBMCs from survivors and non-survivors were treated either with iMSC-EVs (4 µg/ml) or PBS for 24 h. The PBMCs were collected for protein C-reactive protein (CRP, A) and serum amyloid A (SAA, B) by immunofluorescence staining. Scatter bar graph represents mean values and standard error of mean (SE) (n=4/group). S, Survival; NS, Non-Survival.

Serum amyloid A (52) and C-reactive protein (CRP) are acute phase proteins whose production is stimulated by inflammatory cytokines and serve as biomarkers in sepsis. PMBCs from survivors and non-survivors were treated ± iMSC-EVs, SAA and CRP levels were measured by immunofluorescence (**Figure 5**). SAA and CRP protein levels were higher in PMBCs from survivors compared to non-survivors (**Figure 5**, P<0.05). Treatment with iMSC-EVs significantly reduced CRP and SAA levels in PBMCs from survivors, whereas significant increases were observed in PBMCs from non-survivors.

## 4 Discussion

Sepsis is a life-threatening problem characterized by the systemic inflammatory response to infection. The immune response to infection involves both excessive inflammation and compensatory immunosuppression, which together contribute to disease progression and mortality. The current treatment of sepsis includes “source control” of the infection, appropriate antibiotics and supportive care (fluid administration, vasopressors, mechanical ventilation, renal replacement, etc.). The heterogeneous nature of sepsis and its impact on outcomes has been recognized for years. Seymour et al. performed a retrospective analysis of clinical sepsis datasets to better define the different patterns of sepsis seen in clinical studies (53),. They identified four clinical phenotypes of sepsis based on infection type, host response and clinical outcomes (53). With that in mind we focused on patients with surgical sepsis in our study in an effort to recruit a more severely ill, homogeneous population with significant organ failure resembling the δ phenotype in the Seymour study (54). Despite the similarities of our sepsis population to the δ phenotype our patients demonstrated significant heterogeneity in their immune status, higher SOFA scores and mortality than the δ phenotype described by Seymour (53).

The analysis of factors associated with mortality in our study identified a combination of clinical characteristics, cytokine profiles and the immune response of PMBCs to be associated with 60-day mortality. While the clinical parameters of advanced age, SOFA score and cancer diagnosis are logically associated with mortality in sepsis, the biological parameters (cytokine levels/ratios and PMBC immune responses) provide additional insights into the immunomodulatory milieu of the host associated with poor prognosis.

Our findings suggest that circulating levels of anti-inflammatory, immunosuppressive cytokines are associated with mortality. Plasma levels of IL-10 and MCP-1 were significantly increased in non-survivors. Elevated IL-10 levels have been widely associated with immunosuppression and poor outcomes in sepsis, reflecting the host’s compensatory anti-inflammatory response that can impair pathogen clearance and immune competence (3). Similarly, increased MCP-1 has been linked to excessive monocyte recruitment and inflammatory dysregulation in septic patients (55).

The ratio of inflammatory to anti-inflammatory cytokines in plasma was also associated with mortality. The IL-10/TNF ratio was significantly increased and the IL-6/IL-10 ratio was significantly decreased in non-survivors. Previous studies have demonstrated that elevated IL-10 relative to TNF-α is associated with impaired immune competence, reduced pathogen clearance, and increased mortality in septic patients (14, 56, 57). Excessive IL-10 production suppresses macrophage and T-cell activation, while diminished TNF-α signaling may impair early host defense responses, collectively contributing to sepsis-induced immune paralysis (58, 59). Our observation that the rations of IL-6/IL-10 ratio and IL-10/TNF-α were significantly altered between survivals and non-survivors further supports the concept that an imbalance between pro- and anti-inflammatory mediators contributes to disease severity and mortality. Multivariable regression analysis further demonstrated that IL-10, MCP-1, and the IL-10/TNF-α ratio were independently associated with 60-day mortality, whereas the IL-6/IL-10 ratio was inversely associated with mortality risk. These findings are consistent with previous clinical studies indicating that cytokine ratios may better reflect immune status than individual cytokines alone, as they capture the dynamic balance between inflammatory and anti-inflammatory signaling pathways during sepsis progression (60).

Our *in vitro* studies revealed substantial differences in PBMC responses between survivors and non-survivors. Following stimulation with LPS, PBMCs from non-survivors exhibited a more pronounced inflammatory response, including increased IL-10/TNF-α ratios. These findings suggest that immune cells from patients with poor outcomes are characterized by simultaneous activation and immunosuppression. Such immune dysfunction is a hallmark of sepsis and has been described as a state of “persistent inflammation, immunosuppression, and catabolism syndrome” (PICS) (2).

Mesenchymal stromal cells and their extracellular vesicles have emerged as promising therapeutic approaches for modulating immune responses in inflammatory diseases. Increasing evidence suggests that mesenchymal stromal cells exert their therapeutic effects largely through paracrine signaling mediated by extracellular vesicles, which contain bioactive proteins, lipids, and nucleic acids capable of regulating immune cell function (61). In the present study, treatment with iMSC-EVs significantly modulated inflammatory responses in PBMCs derived from septic patients. Interestingly, EV treatment induced immune activation under baseline conditions but suppressed excessive inflammatory responses following LPS stimulation, suggesting that EVs may exert context-dependent immunoregulatory effects. Importantly, iMSC-EVs significantly attenuated LPS-induced immune dysregulation in PBMCs. Co-treatment with iMSC-EVs reduced inflammatory cytokine production and normalized cytokine ratios, particularly in PBMCs from non-survivors (62). Notably, the IL-10/TNF-α ratio decreased following EV treatment in the non-survivor group, suggesting a partial reversal of sepsis-associated immunosuppression (59). These findings support the concept that MSC-derived EVs can restore immune homeostasis by balancing pro- and anti-inflammatory pathways (63). Similar immunomodulatory effects of MSC-EVs have been reported in experimental models of sepsis and acute respiratory distress syndrome (64), where EVs reduced inflammatory cytokine production and improved survival (65–67).

Our findings are consistent with recent transcriptomic analyses that identified five distinct mechanistic endotypes in early sepsis (Neutrophilic-Suppressive, Inflammatory, Innate-Host-Defense, Interferon, and Adaptive), each defined by unique gene-expression signatures and associated with markedly different severity and mortality profiles, our findings on IL-10/TNF-α and IL-6/IL-10 ratios in septic PBMCs further illustrate how iMSC-EVs may help restore immune balance across these heterogeneous patient subgroups (68).

Another important finding of our study is that iMSC-EVs reduced apoptosis in PBMCs from septic patients. Apoptosis of immune cells is a critical feature of sepsis-induced immunosuppression and has been associated with poor outcomes (59, 60, 69). We observed that PBMCs from non-survivors exhibited higher levels of apoptotic cells and increased expression of cleaved caspase-3 (70), consistent with previous reports of extensive lymphocyte apoptosis in severe sepsis (71). Treatment with iMSC-EVs significantly reduced apoptosis and caspase-3 activation in PBMCs, suggesting that EV-mediated signaling may protect immune cells from programmed cell death and preserve immune competence during septic inflammation. In addition to cytokine modulation and anti-apoptotic effects, iMSC-EVs also influenced acute-phase inflammatory markers. Patients with sepsis have elevated SAA levels, which are stronger predictive markers of sepsis severity (72), and CRP similarly rises rapidly as a key indicator of systemic inflammation and infection (73, 74). PBMCs from non-survivors exhibited higher baseline levels of C-reactive protein and Serum amyloid A, both of which are key mediators of systemic inflammation and predictors of sepsis severity (75). Interestingly, EV treatment reduced CRP and SAA levels in PBMCs from survivors but increased these markers in non-survivors. These findings suggest that the immunomodulatory effects of EVs may depend on the immune status or stage of sepsis, highlighting the complexity of immune regulation in critically ill patients (62, 63).

Building upon transcriptomic, metabolomic, and cellular-function studies that have delineated sepsis endotypes with divergent clinical outcomes and differential responses to immunomodulation, the present data position iMSC-EVs as a biologically rational approach capable of restoring immune homeostasis across both hyperinflammatory and immunosuppressive phenotypes (76).

Several limitations should be considered when interpreting these findings. First, this was a single-center exploratory study with a relatively modest sample size (n = 53), which may limit statistical power and the generalizability of the observed associations. Although statistically significant relationships were identified between clinical variables (e.g., age, malignancy, SOFA score) and inflammatory mediators (e.g., IL-10, MCP-1, IL-10/TNF-α ratio) as well as 60-day mortality, these findings require validation in larger, prospective, multicenter cohorts prior to clinical translation. Second, the study population was restricted to surgical sepsis patients, without inclusion of non-septic surgical controls or a comparator cohort of medically managed septic patients with comparable illness severity. This limitation precludes direct mechanistic and immunological comparisons across clinically distinct sepsis phenotypes. While this focus reflects the primary objective of characterizing immune dysregulation and evaluating iMSC-EV–mediated immunomodulation in a surgically defined sepsis population, characterized by operative injury, source control interventions, and postoperative immune perturbation, broader comparative cohorts would have strengthened the interpretive framework. Third, blood samples were obtained within 6 hours of sepsis diagnosis, capturing only a single early postoperative timepoint. The absence of serial sampling limits the ability to characterize longitudinal immune trajectory, including transitions between hyperinflammatory and immunosuppressive phases, as well as the durability of iMSC-EV–associated immunomodulatory effects. Fourth, for certain analytes, including CRP and serum amyloid A, the effective sample size was smaller due to assay availability and sample constraints, which may reduce robustness for these specific measurements. Fifth, the inclusion of patients with do-not-resuscitate (DNR) orders may introduce heterogeneity in treatment intensity, potentially confounding associations between immunological parameters and 60-day mortality. Future studies should prospectively capture and stratify by code status to minimize this source of bias. Sixth, the *in vitro* PBMC experiments, while mechanistically informative, cannot fully recapitulate the complexity of the *in vivo* septic immune microenvironment, including multicellular interactions, endothelial contributions, and dynamic pathogen-host responses. Finally, although our findings demonstrate that iMSC-derived extracellular vesicles exert significant immunomodulatory effects on PBMCs, the precise molecular cargo and signaling pathways responsible for these effects remain incompletely defined and warrant further investigation in future mechanistic studies.

In conclusion, our study demonstrates that inflammatory mediator profiles differ significantly between survivors and non-survivors with sepsis and that iMSC-EVs derived from human induced pluripotent stem cells exert immunomodulatory and cytoprotective effects on PBMCs derived from septic patients. These findings suggest that iMSC-EVs may represent a promising cell-free therapeutic strategy for restoring immune balance and improving outcomes in sepsis. Further studies are necessary to elucidate the molecular mechanisms underlying EV-mediated immune regulation and to evaluate their therapeutic potential in clinical settings.

## Data Availability

The raw data supporting the conclusions of this article will be made available by the authors, without undue reservation.

## Supplementary material

Supplementary Figure 1 Detection of uptake of iMSC-EVs in PBMCs. The iMSC-EVs were labeled using ExoGlowTM-Protein EV Labeling Kit (Green) from SBI. PBMCs were obtained from septic patients. iMSC-EVs uptake in PBMCs were detected by incubation of the cells and labeled iMSC-EVs for 4 h.
