# Supplementary figures and images for: Mesenchymal stem cell - derived extracellular vesicles modulate immune function in sepsis
Source: Front Immunol. 2026 Jul 14;17:1881925. doi: 10.3389/fimmu.2026.1881925 (PMC13407162; doi:10.3389/fimmu.2026.1881925)

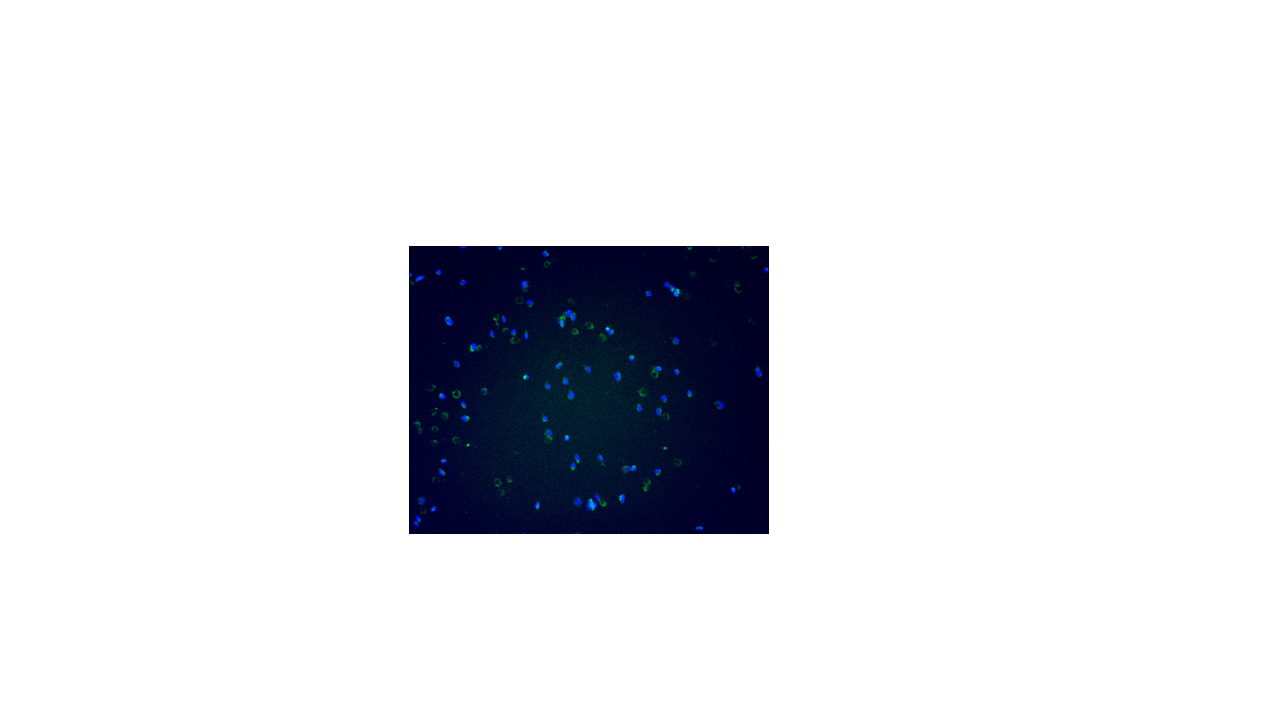

Supplement: Supplementary Figure 1 — Detection of uptake of iMSC-EVs in PBMCs. The iMSC-EVs were labeled using ExoGlowTM-Protein EV Labeling Kit (Green) from SBI. PBMCs were obtained from septic patients. iMSC-EVs uptake in PBMCs were detected by incubation of the cells and labeled iMSC-EVs for 4 h. [file Image1.tif]
