# Supplementary material for: Mesenchymal stem cell - derived extracellular vesicles modulate immune function in sepsis
Source: Front Immunol. 2026 Jul 14;17:1881925. doi: 10.3389/fimmu.2026.1881925 (PMC13407162; doi:10.3389/fimmu.2026.1881925)
Supplement: Supplementary file 2 [file Table1.docx]

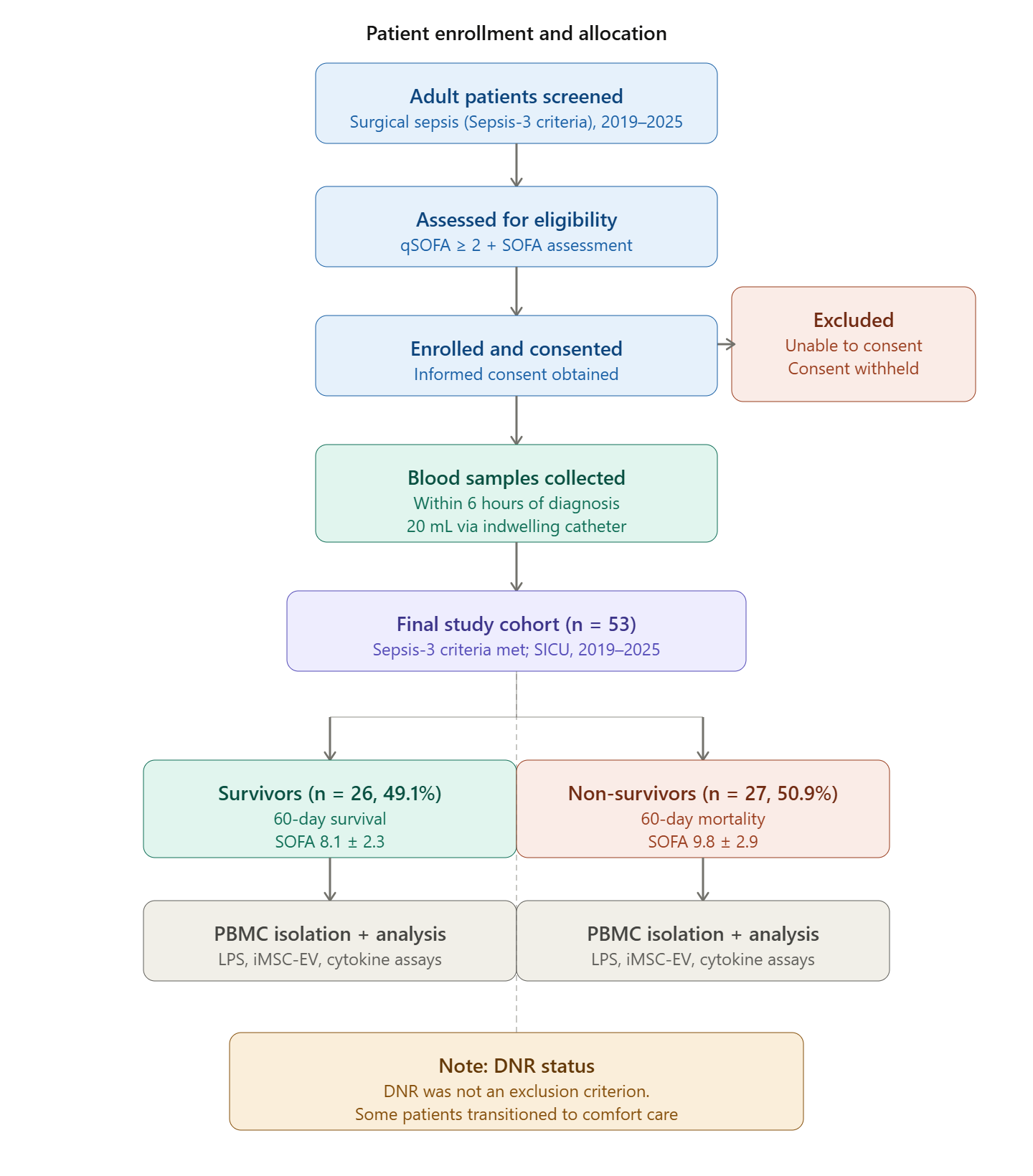


**Supplemental Figure 2. Patient enrollment and allocation flowchart**. Adult patients with suspected surgical sepsis were screened using Sepsis-3 criteria and qSOFA ≥ 2 between 2019 and 2025 at the SICU of SUNY Upstate Medical University. Patients were excluded only if informed consent could not be obtained. A total of 53 consecutively enrolled patients comprised the final cohort, stratified by 60-day outcome into survivors (n = 26) and non-survivors (n = 27). Peripheral blood mononuclear cells (PBMCs) from both groups underwent ex vivo LPS stimulation and iMSC-EV treatment with cytokine, apoptosis, and acute-phase protein analyses. DNR status was not an exclusion criterion; some patients were transitioned to comfort care during the study period.
